# Selenium as a Factor Moderating Depression and Obesity in Middle-Aged Women

**DOI:** 10.3390/nu15071594

**Published:** 2023-03-25

**Authors:** Daria Schneider-Matyka, Anna Maria Cybulska, Małgorzata Szkup, Bogumiła Pilarczyk, Mariusz Panczyk, Anna Lubkowska, Nikola Sadowska, Elżbieta Grochans

**Affiliations:** 1 Department of Nursing, Pomeranian Medical University in Szczecin, Żołnierska Str. 48, 71-210 Szczecin, Poland; 2 Department of Animal Reproduction Biotechnology and Environmental Hygiene, West Pomeranian University of Technology, Klemensa Janickiego Str. 29, 71-217 Szczecin, Poland; 3 Department of Education and Research in Health Sciences, Faculty of Health Sciences, Medical University of Warsaw, Litewska Str. 14/16, 00-581 Warsaw, Poland; 4 Department of Functional Diagnostics and Physical Medicine, Pomeranian Medical University in Szczecin, Żołnierska Str. 54, 71-210 Szczecin, Poland

**Keywords:** selenium, depression, obesity, middle-aged women, cytokines

## Abstract

The aim of this study was to evaluate the effect of serum selenium on PPAR-γ and the selected proinflammatory cytokines (IL-1β, IL-6, TNF-α) in relation to depressive symptoms and obesity in middle-aged women. The research procedure was as follows: a survey was performed using the authors’ questionnaire and the BDI, anthropometric measurements, and the analysis of blood for the levels of selenium, cytokines, and genetic analysis of the PPAR-γ polymorphism (*n* = 443). It was found that the BMI increased along with the concentration of IL-6. No moderating effect of selenium was observed, although the cut-off values for “*p*” were established for IL-β*Se (*p* = 0.068) and IL-6*Se (*p* = 0.068), so there was a potential association with these two markers. At high selenium levels, the effect of higher IL-β levels on a decrease in BMI was stronger, as was the effect of an increase in IL-6 levels on an increase in BMI. No effect of selenium on PPAR-γ was found in relation to depressive symptoms and obesity. Higher selenium levels may have a beneficial effect on BMI even at high IL-β concentrations, however, at high IL-6 concentrations, this effect was not observed. Selenium levels had no impact on depressive symptoms.

## 1. Introduction

Selenium (Se) is a metalloid or semi-metal—a chemical element that has properties of both metal and non-metal. Discovered in 1817, it was recognized as an essential trace element in higher animals by K. Schwarz and C. M. Foltz in 1957 [1]. Additionally, the WHO has confirmed the role of selenium as an essential trace element [2]. Selenium is absorbed from food in inorganic (selenite, selenate) and organic (selenomethionine, selenocysteine) forms. It is metabolized to selenide (Se^2−^), and then to selenophosphate (SeH_3_PO_3_), which is incorporated into proteins through a unique tRNA pathway [3,4].

Many biological properties of selenium result from its role as a component of selenium-containing proteins (selenoproteins), where it is often found in the form of selenocysteine in the active site of the enzyme [5]. Selenium is incorporated into selenoproteins as the 21st amino acid—selenocysteine [6]. Selenocysteine is present in at least 25 human selenoproteins involved in a wide range of basic biological functions, from the regulation of reactive oxygen species (ROS) to hormone biosynthesis [7]. The first and best characterized of the selenoproteins is cytosolic glutathione peroxidase (GPx-1), whose activity may affect cellular signaling pathways [8]. Selenoprotein activity is optimal when the plasma selenium levels are adequate. The WHO recommends a selenium intake of at least 55 g/day for adults. At the same time, it should be noted that selenium is characterized by a narrow range of therapeutic concentrations, and that adverse health effects occur in both the deficiency and excess of this element [9]. The most widely documented selenium deficiency in humans is Keshan disease, which manifests, among others, as changes in the heart and other striated muscles. Another health problem is degenerative joint disease, which mainly affects young children (Kashin–Beck disease). On the other hand, when selenium concentrations are too high (with a daily intake of more than 1000 μg), there is a risk of selenium poisoning. The symptoms include gastrointestinal irritation, nail and skin lesions, hair loss and, in severe cases, nerve damage [10,11]. Selenoproteins have been shown to regulate the functioning of the endocrine system and intracellular signaling [12]. It is now recognized that normal selenium concentrations are essential for the immune [13], endocrine [14], cardiovascular [15], reproductive [6], and nervous systems [16]. Selenium deficiency may be associated with an increased risk of cancer and cardiovascular diseases [9,17]. Its deficiency or excess may lead to a variety of diseases, such as mental disorders and obesity [18,19]. Dietary selenium intake is considered a key determinant of selenium levels. Products contributing to daily selenium intake include Brazil nuts, meat and offal (liver and kidney), garlic, onion, broccoli, and cauliflower [20]. It has been found that the gut microbiota may affect the metabolism of selenoproteins [21]. Some internal factors causing changes in the intestinal microflora can also modify the bioavailability of selenium in the diet, and its concentration, particularly in inflammatory bowel disease, Crohn’s disease, colorectal cancer, and metabolic diseases (e.g., obesity) [21,22]. The role of selenium in obesity has not so far been fully explained. The data coming from observational studies on the relationship of selenium with obesity [23] and metabolic syndrome (MetS) [24] are contradictory. However, high selenoprotein expression has been demonstrated in adipose tissue in both healthy [25] and obese [26] individuals, which may indicate an important role of selenium in adipocyte biology. 

Peroxisome proliferator-activated receptor gamma (PPAR-γ) belongs to the nuclear hormone receptor family. It is an important mediator in the regulation of metabolic processes [27]. The research findings on PPAR-γ suggest that it is essential for lipid metabolism. It takes part in the process of adipogenesis, and so it is an important parameter in obesity research. It is partly responsible for maintaining the serum glucose homeostasis. Activated PPAR-γ positively affects the expression of the glucose transporter 4 (GLUT4) in adipocytes, which has a beneficial effect on glucose transport. PPAR-γ plays a part in insulin resistance—it improves insulin sensitivity, thereby exerting an opposite effect to TNF-α. The presence of PPAR-γ in endothelial cells and vascular smooth muscle cells may be important for the regulation of inflammation and atherosclerosis. It also plays a role in carcinogenesis [28,29,30,31]. PPAR-γ has been shown to have antidepressant-like effects [32].

Both in vivo and in vitro studies show that selenate and selenite have an indirect effect on adipogenesis. Selenium may protect against obesity by modulating the PPAR signaling and developing lipophilic selenium compounds capable of binding PPAR [33,34].

Selenium seems to have the ability to alleviate inflammatory signaling pathways [9,35]. Obesity is associated with chronic low-grade inflammation. Depression is also defined as an inflammatory disorder. The levels of inflammatory cytokines (including TNF-α, IL-1β)—that are parameters associated with obesity—are also related to the severity of depression [36,37]. Elevated levels of TNF-α have been found in depression and manic states [38,39], bipolar disorder [40], and obesity [41]. In overweight and obese adults, plasma levels of tumor necrosis factor alpha (TNF-α) and interleukin 6 (IL-6) were significantly increased [42,43,44]. It was also found that a high-fat diet (HFD) in piglets significantly reduced the expression of selenoproteins in lymphocytes (except for Gpx1, Gpx2, SelT, and SelM), and this effect was exacerbated by low selenium intake, which enhanced the proinflammatory effect of a HFD by up-regulating the expression of TNF, NF-B, IL-1, IL-6, IL-8, and IL-9 [45]. 

The perimenopausal period, which falls between the ages of 45 and 65, has been identified as a high-risk stage for weight gain (half a kilogram weight gain per year is observed). Midlife weight gain is a consequence of low levels of circulating estrogen due to the progressive loss of ovarian function. Changes in the hormonal environment, chronological aging, a decline in physical activity combined with a Western diet, and recurrent emotional eating episodes associated with psychological distress also contribute to putting on weight. A slow metabolic rate in middle-aged women reduces the ability to burn calories efficiently, promoting a positive caloric balance [46]. Depression is a significant public health problem worldwide. In middle-aged women, depression may impair the quality of life, sleep, sexual function, immunity, and life satisfaction. It is also associated with a higher risk of other health problems, such as poor cognitive function, cardiovascular disease [47], osteoporosis [48,49], and metabolic syndrome [50]. Studies confirm an increased risk of depressive symptoms in middle-aged women [51,52,53,54]. Therefore, it seems reasonable to consider the influence of selenium, PPAR-γ, and selected proinflammatory cytokines in the context of obesity and depression among middle-aged women. 

The aim of this study was to evaluate the effect of serum selenium on PPAR-γ and the selected proinflammatory cytokines (IL-1β, IL-6, TNF-α) in relation to depressive symptoms and obesity in middle-aged women.

## 2. Materials and Methods

The study sample included 443 middle-aged women living in north-western Poland. Recruitment was based on advertisements in the local press and information posters in public places. All participants were residents of the West Pomeranian Voivodeship (Poland). The criteria for inclusion in the study were:

- Female sex;
- No selenium supplementation;
- No inflammatory, psychiatric or cancerous disease;
- No alcohol abuse.

The norm for alcohol consumption was less than 20 g of pure alcohol per day or occasionally no more than 40 g of pure alcohol, and declaring at least two days of abstinence from alcohol per week State Agency for Solving Alcohol Problems [55]. Only women who met all of the above criteria were included in the study. 

The research procedure consisted of three parts. The first part was based on a survey performed using a questionnaire by the authors and The Beck Depression Inventory (BDI).

- The authors’ questionnaire concerned:
  - Sociodemographic data;
  - Selected medical information on chronic diseases, medications taken for hypertension, hypertriglyceridemia, hyperglycemia and too high HDL levels;
  - Addiction to cigarettes and alcohol.
- The Beck Depression Inventory (BDI) is a scale used to self-assess the presence and severity of depressive symptoms. Interpretation of the results was based on the following criteria:
  - 0–11 points meant no depressive symptoms;
  - 12–26 points meant mild depressive symptoms;
  - 27–49 points meant moderate depressive symptoms;
  - 50–63 points suggested severe depressive symptoms [56,57].

In the second part of the study, anthropometric measurements were taken. A Tanita MC780 MA (Poznań, Poland) device was used to assess individual body composition. The mass of body fat (MBF) in kilograms and the percent of body fat (PBF), as well as the amount of visceral fat were measured. The norm for the PBF for women aged 40–59 was 23% to 34%, and for women aged 60–79 was 24–36%. The normal values for visceral adipose tissue ranged from 1 to 12 [58]. The body weight and height were also measured. The body mass index (BMI) was calculated using the formula: weight in kilograms divided by height in meters squared (kg/m^2^). The following norms for BMI were adopted:

- 18.5–24.9—correct weight to height ratio;
- below 18.5—underweight;
- 25.0–29.9—overweight;
- above 30.0—obesity [59].

In the third part of the study, biological material (blood) was collected from a peripheral vein in accordance with the procedure for collecting, storing, and transporting biological material. The blood was collected between 7:00 a.m. and 9:30 a.m. after an overnight fast and 10 min rest in a sitting position. The biological material was collected into two Vacutainer tubes (Sarstedt, Nümbrecht, Germany): the first one with 1 g/L K2, ethane-1,2-diyl dinitrilotetraacetic acid, and the second one for serum biochemical analysis (7 mL). 

Selenium, IL-1β, IL-6, and TNF-α levels were measured. Selenium concentrations between 93–121 µg/L were regarded as reference values. Selenium levels were determined by the spectrofluorimetric method using 2,3-diaminonaphthalene (Shimadzu RF-5001 PC, Kyoto, Japan). The samples were subjected to wet digestion in a mixture of concentrated acids HNO3 (230 °C, 180 min) and HClO4 (310 °C, 20 min). The measurement was performed at an emission wavelength of 518 nm and an excitation wavelength of 378 nm. 

The assumed norms were: 15–500 pg/mL for IL-1β, 2–200 pg/mL for IL-6 [60], and <16 pg/mL for TNF-α. Serum levels of IL-1β, IL-6, and TNF-α were measured by enzyme immunoassays using commercially available ELISA kits (DRG Diagnostics, Marburg, Germany) according to the manufacturer’s protocol. The sensitivity of the IL-1β assay was 0.35 pg/mL, and the intra- and inter-assay CVs were 2.3% and 4.9%, respectively. The sensitivity of the IL-6 assay was 2 pg/mL, the intra- and inter-assay CVs were 4.2% and 4.4%, respectively. The sensitivity of the TNF-α assay was 0.7 pg/mL, the intra- and inter-assay CVs were 6.3% and 4.5%, respectively. 

DNA was then isolated for genetic analysis of the PPAR-γ rs1801283 polymorphism. Genomic DNA was isolated from whole blood in accordance with standard procedures. Genotyping was based on the real-time fluorescence resonance energy transfer performed using the Light Cycler System 1.0 (Roche Diagnostic, Warsaw, Poland). The gene polymorphisms were determined under the following conditions: polymerase chain reaction (PCR) was performed with 50 ng DNA in a total volume of 20 mL containing 2 mL reaction mix, 0.5 mM of each primer, 0.2 mM of each hybridization probe, and 2 mM MgCl_2_ for 35 cycles of denaturation (95 °C for 10 min), annealing (60 °C for 10 s), and extension (72 °C for 15 s) as the manufacturer suggests. After amplification, a melting curve was generated by keeping the reaction temperature at 40 °C for 20 s, and then slowly raising it to 85 °C. The fluorescence signal was plotted against the temperature to give a melting curve for each sample. The polymorphisms were determined by analyzing the melting curves. In the PPAR-γ rs1801282 (C > G) polymorphism, peaks were obtained at 53.14 °C for the G allele and at 62.12 °C for the C allele. Biological material for the DNA analysis was stored and transported in accordance with the procedures of the Quality Management System of the Genetic Laboratory, the Department of Psychiatry (according to the EN 15189 standard). 

The study was conducted in accordance with ethical standards and the Declaration of Helsinki. The study protocol was approved by the Bioethics Committee of the Pomeranian Medical University in Szczecin, Poland (KB-0012/181/13). The subjects gave informed written consent to participate in the study.

### Statistical Analysis

The collected data were analyzed using descriptive statistics. The mean (M), standard deviation (SD), minimum and maximum value and the coefficient of variation (CV) for the quantitative variables were calculated. The qualitative variables were presented as numbers (N) and frequencies (%).

Statistical inference was performed using null hypothesis testing. Two-sided *p* < 0.05 was considered statistically significant for all the null hypotheses tested. The relationship between the types of the PPAR-γ rs1801282 polymorphism and the BMI categories or depressive symptoms was assessed using the Kruskal–Wallis H test. The correlation of the selected predictors (Allele C(+) or G(+), Se [mg/L], IL-β [pg/mL], TNF-α [pg/mL], IL-6 [pg/mL]) with the BMI value and the BDI score was estimated using a multivariate regression model. The estimation of the model parameters was calculated using the least squares method. A non-standardized (b) and a standardized (β) regression coefficient with a 95% confidence interval (95% CI) were calculated for each predictor. To determine the effect of the selenium concentration on the correlation between the selected predictors and the BMI value and BDI score, the interaction effect was calculated in a linear regression model.

All statistical calculations were performed using the STATISTICA software version 13.3 (TIBCO Software, version 13.3, Palo Alto, CA, USA). 

## 3. Results

The sociodemographic data are presented below in Table 1. Almost half of the respondents (48.98%) had a university degree, and 38.60% had secondary education. In total, 71.1% of the subjects lived in a big city, 75.40% were in a formal relationship, and 87.36% were employed. Overall, 35.21% were normal weight, 37.70% were overweight, and 27.09% were obese. Most of the respondents (67.49%) admitted to using stimulants—48.31% of them smoked cigarettes and 25.28% consumed alcohol. Alcohol consumption, however, did not exceed the norms [51]. Only 17.83% took dietary supplements, but they did not contain selenium (Table 1).

Table 2 shows the descriptive statistics on age, selenium concentrations, selected anthropometric variables, depressive symptoms, and the proinflammatory cytokines (IL-6, IL-β, TNF-α). The mean age of the subjects was 53.27 ± 5.09. The mean level of selenium was 92.75± 22.10 µg/L. The mean body weight was 73.02 ± 13.60 kg; BMI was 27.49 ± 5.14; MBF was 25.56 ± 8.65 kg; PBF was 34.08%. The mean score for depressive symptoms as measured by the BDI was 7.03 ± 6.86. The mean IL-6 concentration was 39.74 ± 109.28 pg/mL; IL-6 was 129.50 ± 246.80 pg/mL; TNF-α was 5.47 ± 9.05 pg/mL. In most cases, the mean values of the variables were within normal limits, except for the BMI (the upper limit of normal for the BMI is 24.9, and the mean BMI value was 27.49). In the case of the PBF, the upper limit of normal for women aged 40–59 years is up to 34%, and for women aged 60–79 years is up to 36%—the mean values of this variable were within the upper limit of normal and amounted to 34.08% (Table 2).

The distribution of the genotypes and alleles of the PPAR-γ rs1801282 polymorphism was also described. The CC genotype was found in 69.75%, CG in 25.28%, and GG in 4.97% of the subjects. The C allele was carried by 95.03%, and the G allele by 30.25% (Table 3).

In the further part of the study, the influence of particular genotypes of the PPAR-γ rs1801282 polymorphism on the BMI and depressive symptoms was analyzed. No statistically significant differences were demonstrated (Table 4).

Next, a logistic regression model was used to assess the odds of having an above-normal BMI and depressive symptoms depending on the PPAR-γ with regard to the role of selenium (with and without moderation). The analysis showed no statistically significant relationships (Table 5, Table 6, Table 7 and Table 8). 

It was then found that none of the factors alone affected the BMI, although a cut-off value of *p* = 0.052 was established for IL-6. 

A logistic regression model was used to assess the odds of having an above-normal BMI depending on the levels of the proinflammatory cytokines (IL-1β, IL-6, TNF-α), with and without the effect of selenium and the PPAR-γ alleles (Table 9). 

It was found that higher levels of IL-6 were accompanied by higher BMI values (Table 10). No effect of selenium was observed, although the cut-off values for “*p*” were established for IL-β*Se (*p* = 0.068) and IL-6*Se (*p* = 0.068), so there was a potential moderating effect for these two markers. The BMI was found to decrease with an increase in the level of IL-β, but this relationship varied between the groups depending on the levels of selenium (which was a potential moderator). At high selenium levels, an increase in the concentration of IL-β had a stronger effect on a decrease in the BMI. Elevated IL-6 levels were accompanied by higher BMI values, but again, this relationship varied between the groups depending on the level of selenium. At high selenium levels, an increase in the concentration of IL-6 had a stronger effect on an increase in the BMI (Table 11). The alleles did not have a moderating effect (Table 12).

None of the factors alone affected depressive symptoms, however a trend value of *p* = 0.077 was noted for IL-β (Table 13). 

A logistic regression model was also used to assess the odds of depressive symptoms depending on the levels of the proinflammatory cytokines (IL-1β, IL-6, TNF-α), both with and without the effect of selenium and the PPAR-γ alleles. The influence of selenium and the alleles was not confirmed (Table 14, Table 15 and Table 16).

## 4. Discussion

Selenium is one of the micronutrients that is particularly important for the functioning of the human body [9,61,62]. Poland, however, is a region with low selenium intake [63], so there is a risk of its widespread deficiency, resulting in reduced selenoprotein activity [11]. By regulating the activity of selenoproteins, selenium influences the processes that play a key role in the prevention and modulation of risk factors for many diseases, including cancer, diabetes, Alzheimer’s disease, mental disorders, cardiovascular disorders, fertility disorders, inflammation, and infections [7,64]. 

In our investigation, the mean serum selenium concentration was 92.75 ± 22.10 µg/L, which was below the lower reference limit (93–121 µg/L). Other studies conducted in the Polish healthy adult population also show low serum selenium levels, which may be related to the low content of this element in the soil [65]. In a study by Hać et al., the mean plasma selenium concentration was 73.3 ± 14.1 μg/L (76.7 ± 13.2 μg/L in men; 70.4 ± 14.7 μg/L in women). Furthermore, 20% of the subjects had plasma selenium levels lower than 60 μg/L [66]. Additionally, Kłapcińska et al. reported a relatively low mean selenium concentration (62.5 ± 18.4 μg/L), with lower selenium levels observed in women (57.5 ± 18.9 μg/L) than in men (65.9 ± 17.2 μg/L). In 40% of healthy adults, selenium levels ranged from 60 to 80 μg/L, which means that they were below the lower reference limit [67]. Similarly, in another study by Kłapcińska et al., the mean selenium level was low (63.5 ± 18.1 μg/L), and again selenium concentrations were significantly lower in women (58.1 ± 18.9 μg/L) than in men (66.3 ± 17.0 μg/L), (*p* < 0.01) [68]. In a study by Kluza et al., the mean concentration of selenium in the group of healthy controls was 78.99 g/L [69].

PPAR-γ is significantly involved in a signaling pathway that occurs at the inter-section of the depression and obesity pathways. Selenium, on the other hand, in addition to its anti-inflammatory, anticancer, and antioxidant effects, may also affect PPAR-γ. In our study, a logistic regression model was used to assess the odds of having an elevated BMI and depressive symptoms depending on PPAR-γ. Then, using the logistic regression model, the impact of PPAR-γ on the aforementioned variables was assessed, taking into account the influence of selenium—the study, however, showed no significant relationships. 

According to other authors, the activation of PPAR-γ relieves symptoms associated with cognitive dysfunctions and obesity, and improves the level of brain-derived neurotrophic factor (BDNF), which is reduced in depression and type 2 diabetes [9,70]. Additionally, selenium has been shown to have the ability to attenuate inflammatory signaling pathways [9,35]. Other studies indicate that element supplementation (Zn, Mg, Se) enhances the effect of antidepressants or has an antidepressant effect itself [71]. Both PPAR-γ and selenium are parameters that may lead to changes associated with obesity and mood disorders. PPAR-γ is found in high amounts in hippocampal neurons and in areas that play an important role in depression [19,72,73,74]. 

In a study by Zhong et al., which involved 6440 men and 6849 women, serum selenium levels negatively correlated with BMI. A high percent of body fat (PBF) was accompanied by lower serum selenium levels [75]. Alasfar et al. reported that selenium levels were significantly lower in about 15% of women with severe obesity compared to slim women from the control group [76]. According to Błażewicz et al., selenium may play a role in obesity (in their study, obese children had lower selenium levels) [19]. Another investigation conducted in the general Japanese population showed that the plasma selenium levels were directly related to waist circumference, but not to the BMI [77].

Higher selenium intake has been found to be associated with lower BMI, smaller waist circumference, and lower body fat content in both women and men [78]. On the other hand, research on the effect of consuming selenium-rich Brazil nuts did not demonstrate any changes in the plasma levels of inflammatory biomarkers (IL-6, TNF-α, and toll-like receptors 2 and 4) in obese women. However, there was a significant increase in gene expression, suggesting that eating Brazil nuts is a proinflammatory stimulus in obesity [79]. 

The results of our study indicated that as the concentration of IL-6 increased, so did the BMI. In our further analysis of the effect of selenium, we found cut-off values for *p* in the case of IL-1β and IL-6, so it can be assumed that selenium had a potential effect on these two markers. Our study showed that BMI decreased with an increase in IL-β concentrations if the level of selenium was high—the effect of an increase in IL-β concentration on a decrease in BMI was stronger than at low selenium levels. At the same time, at high selenium levels, BMI increased along with the concentration of IL-6—the effect of elevated IL-6 levels on an increase in BMI was stronger than at low selenium concentrations. 

Our findings have not confirmed the effect of selenium in the context of depressive symptoms, probably due to the lack of depressive symptoms or mild depressive symptoms in the vast majority of the respondents (98.7%). Nevertheless, numerous studies indicate a link between selenium and depressive symptoms. An organic diet containing selenium has been found to improve cognitive functions and bring positive changes in the psychoemotional state (reduced anxiety and emotional instability) [80]. Optimal serum selenium levels reduce the risk of depressive symptomatology [81]. High selenium levels in groundwater are associated with fewer depressive symptoms [82] and lower scores on The Geriatric Depression Scale (GDS) [83]. Conversely, low dietary selenium intake entails a higher risk of de novo major depression disorders (MDD) [84]. Using selenium supplements protects against the symptoms of postpartum depression [85]. TNF-α has been shown to play a key role in the pathophysiology of depression. Nuclear factor kappa B (NF-κB) and p38 mitogen-activated protein kinase (MAPK) are important proteins in the signaling activated by TNF-α. They are found in the prefrontal cortex and hippocampus, which are important brain areas involved in the antidepressant response. TNF-α raises the levels of NF-κB and p38 [86]. Selenium reduces the proinflammatory expression of TNF-α by inhibiting NF-κβ and p38 MAPK [87]. In a study by Gandhi et al., selenium was also shown to down-regulate NF-κβ with a corresponding increase in the PPAR-γ activation [35]. 

The study presented here has some limitations. The study sample included mainly healthy respondents. Although they were middle-aged women, who are more likely to develop depressive disorders, the majority of them did not suffer from depressive symptoms, which may have affected the results of the study. It would be reasonable to continue the research among women with depressive disorders. It is also noteworthy that most of the respondents had normal levels of selenium and proinflammatory cytokines (IL-1β, IL-6, TNF-α). For broader analysis of the relationships between selenium and IL-1β, IL-6, and TNF-α, the values of the above-mentioned variables exceeding the normal limits should be used as criteria for inclusion in the study. 

Recruitment of the participants was carried out through information leaflets and posters distributed in public places, and advertisements in the local press. However, despite our efforts, there is a risk that the respondents did not constitute a representative sample. 

## 5. Conclusions

1. No effect of selenium on PPAR-γ was found in relation to depressive symptoms and obesity among middle-aged women.
2. Higher selenium levels may have a beneficial effect on BMI even at high IL-β concentrations, however, at high IL-6 concentrations, this effect was not observed.
3. Selenium levels had no impact on depressive symptoms among the subjects.

## Figures and Tables

**Table 1:** Characteristics of the study sample (*N* = 443).

	*n*	%
Education		
primary	13	2.93
vocational	42	9.48
secondary	171	38.60
third-level	217	48.98
Place of residence		
village	48	10.84
small city	80	18.06
big city	315	71.10
Marital status		
single	77	17.38
informal relationship	32	7.22
formal relationship	334	75.40
Employment status		
unemployed	56	12.64
employed	387	87.36
Age		
≤45	25	5.64
46–50	119	26.86
51–55	142	32.05
56–60	126	28.44
61–65	29	6.55
>65	2	0.45
BMI category		
normal weight (<25.00)	156	35.21
overweight (25.00–29.99)	167	37.70
obesity (≥30.00)	120	27.09
Using stimulants	299	67.49
Cigarettes	214	48.31
Alcohol	112	25.28
Supplements	79	17.83

**Table 2:** Characteristics of the study sample (*N* = 443).

Parameter	M	SD	Min	Max	CV [%]
Age (years)	53.27	5.09	43.00	66.00	9.55
Selenium (mg/L)	92.75	22.10	34.78	178.50	23.83
Weight (kg)	73.02	13.60	16.30	123.40	18.63
BMI (kg/m^2^)	27.49	5.14	17.50	47.50	18.70
MBF (kg)	25.56	8.65	6.00	56.60	33.85
PBF (%)	34.08	6.04	10.20	48.60	17.73
Visceral fat	9.71	4.34	1.00	20.00	44.73
BDI score	7.03	6.86	0.00	38.00	97.59
IL-6 [pg/mL]	39.74	109.28	0.78	864.40	274.99
IL-β [pg/mL]	129.50	246.80	0.34	998.30	190.58
TNF-α [pg/mL]	5.47	9.05	0.01	81.10	165.44

M—mean; SD—standard deviation; CV—coefficient of variation; Min—minimum value; Max—maximum value; MBF—mass of body fat; PBF—percent of body fat.

**Table 3:** Distribution of the genotypes and alleles of the PPAR-γ rs1801282 polymorphism in the study sample (*N* = 443).

	*n*	%
Genotypes		
CC	309	69.75
CG	112	25.28
GG	22	4.97
Allele C		
(−)	22	4.97
(+)	421	95.03
Allele G		
(−)	309	69.75
(+)	134	30.25

**Table 4:** Influence of particular genotypes of the PPAR-γ *rs1801282* polymorphism on BMI and depressive symptoms.

Parameter	PPAR-γ CC		PPAR-γ CG		PPAR-γ GG		*p*-Value *
	*n*	%	*n*	%	*n*	%	
BMI category							
normal	104	33.66	43	38.39	9	40.91	0.349
overweight	115	37.22	44	39.29	8	36.36	
obesity	90	29.13	25	22.32	5	22.73	
Severity of depressive symptoms							
≤11	249	80.58	84	75.00	17	77.27	0.445
12–26	56	18.12	26	23.21	4	18.18	
27–49	4	1.29	2	1.79	1	4.55	
50–63	0	0.00	0	0.00	0	0.00	

* Kruskal–Wallis H test.

**Table 5:** Dependent variable: BMI (without moderation).

	b	β	−95% CI	+95% CI	*p*-Value
Intercept	1.254				<0.001
Allele C (+)	0.000	0.010	−0.090	0.110	0.849
Allele G (+)	−0.001	−0.052	−0.152	0.048	0.304
Se (mg/L)	−0.000	−0.010	−0.104	0.084	0.828

b—unstandardized regression coefficient; β—standardized regression coefficient; CI—confidence interval.

**Table 6:** Dependent variable: BMI (with moderation).

	b	β	−95% CI	+95% CI	*p*-Value
Intercept	1.278				<0.001
Allele C (+)	−0.027	−0.706	−1.657	0.244	0.145
Allele Gv(+)	−0.003	−0.142	−1.143	0.859	0.780
Se [mg/L]	−0.003	−0.151	−0.355	0.053	0.146
Allele C (+)*Se [mg/L]	0.003	0.740	−0.235	1.715	0.136
Allele G (+)*Se [mg/L]	0.000	0.089	−0.908	1.085	0.861

b—unstandardized regression coefficient; β—standardized regression coefficient; CI—confidence interval.

**Table 7:** Dependent variable: the BDI score (without moderation).

	b	β	−95% CI	+95% CI	*p*-Value
Intercept	0.906				0.210
Allele C (+)	0.162	0.049	−0.051	0.149	0.338
Allele G (+)	0.046	0.029	−0.071	0.129	0.567
Se [mg/L]	0.117	0.075	−0.019	0.168	0.119

b—unstandardized regression coefficient; β—standardized regression coefficient; CI—confidence interval.

**Table 8:** Dependent variable: the BDI score (with moderation).

	b	β	−95% CI	+95% CI	*p*-Value
Intercept	2.063				0.177
Allele C (+)	−0.959	−0.289	−1.238	0.660	0.550
Allele G (+)	0.626	0.398	−0.601	1.398	0.434
Se [mg/L]	−0.008	−0.005	−0.209	0.199	0.962
Allele C (+)*Se [mg/L]	0.121	0.353	−0.620	1.326	0.476
Allele G (+)*Se [mg/L]	−0.061	−0.370	−1.365	0.624	0.465

b—unstandardized regression coefficient; β—standardized regression coefficient; CI—confidence interval.

**Table 9:** Influence of the proinflammatory cytokines (IL-1β, IL-6, TNF-α) on BMI with regard to the role of selenium.

Variable	*p*-Value
Allele C	0.5656
Allele G	0.2377
Se	0.8001
TNF-α	0.9440
IL-β	0.1148
IL-6	0.0524

**Table 10:** Model without moderation.

	b	β	−95% CI	+95% CI	*p*-Value
Intercept	1.255				<0.001
Allele C (+)	0.000	0.010	−0.090	0.110	0.848
Allele G (+)	−0.001	−0.048	−0.148	0.051	0.342
Se [mg/L]	−0.000	−0.020	−0.115	0.076	0.688
IL-β [pg/mL]	0.000	0.011	−0.088	0.111	0.821
TNF-α [pg/mL]	−0.003	−0.093	−0.191	0.005	0.064
IL-6 [pg/mL]	0.003	0.104	0.009	0.199	0.033

b—unstandardized regression coefficient; β—standardized regression coefficient; CI—confidence interval.

**Table 11:** Model I with moderation by selenium.

	b	β	−95% CI	+95% CI	*p*-Value
Intercept	1.247				<0.001
Allele C (+)	0.001	0.021	−0.079	0.120	0.682
Allele G (+)	−0.001	−0.055	−0.154	0.045	0.281
Se [mg/L]	0.001	0.030	−0.288	0.349	0.851
IL-β [pg/mL]	0.005	0.502	−0.610	1.613	0.376
TNF-α [pg/mL]	0.030	0.801	−0.171	1.773	0.106
IL-6 [pg/mL]	−0.022	−0.818	−1.815	0.180	0.108
IL-β [pg/mL]*Se [mg/L]	0.003	0.966	−0.073	2.005	0.068
TNF-α [pg/mL]*Se [mg/L]	−0.001	−0.504	−1.645	0.636	0.385
IL-6 [pg/mL]*Se [mg/L]	−0.004	−0.929	−1.927	0.070	0.068

b—unstandardized regression coefficient; β—standardized regression coefficient; CI—confidence interval.

**Table 12:** Model II with moderation by the alleles.

	b	β	−95% CI	+95% CI	*p*-Value
Intercept	1.248				<0.001
Allele C (+)	−0.009	−0.228	−0.574	0.118	0.197
Allele G (+)	0.000	0.020	−0.337	0.377	0.914
Se [mg/L]	−0.000	−0.019	−0.115	0.077	0.699
IL-β [pg/mL]	0.001	0.116	−0.132	0.365	0.359
TNF-α [pg/mL]	0.001	0.018	−0.211	0.247	0.881
IL-6 [pg/mL]	0.004	0.140	−0.116	0.395	0.284
Allele C*IL-β [pg/mL]	0.001	0.149	−0.144	0.441	0.318
Allele G*IL-β [pg/mL]	0.001	0.078	−0.097	0.253	0.380
Allele C*TNF-α [pg/mL]	0.005	0.190	−0.150	0.529	0.273
Allele G*TNF-α [pg/mL]	0.001	0.072	−0.205	0.349	0.609
Allele C*IL-6 [pg/mL]	0.001	0.033	−0.341	0.408	0.862
Allele G*IL-6 [pg/mL]	−0.001	−0.110	−0.378	0.158	0.422

b—unstandardized regression coefficient; β—standardized regression coefficient; CI—confidence interval.

**Table 13:** Influence of the proinflammatory cytokines (IL-1β, IL-6, TNF-α) on depressive symptoms with regard to the effect of selenium.

Variable	*p*-Value
Allele C	0.8337
Allele G	0.6153
Se	0.3129
TNF-α	0.0770
IL-β	0.1098
IL-6	0.4630

**Table 14:** Model without moderation.

	b	β	−95% CI	+95% CI	*p*-Value
Intercept	3.619				0.307
Allele C (+)	0.225	0.014	−0.086	0.114	0.780
Allele G (+)	0.170	0.023	−0.077	0.123	0.655
Se [mg/L]	0.275	0.037	−0.059	0.133	0.449
IL-β [pg/mL]	0.296	0.067	−0.033	0.167	0.188
TNF-α [pg/mL]	0.993	0.063	−0.035	0.162	0.206
IL-6 [pg/mL]	−0.653	−0.059	−0.154	0.037	0.229

b—unstandardized regression coefficient; β—standardized regression coefficient; CI—confidence interval.

**Table 15:** Model I with moderation by selenium.

	b	β	−95% CI	+95% CI	*p*-Value
Intercept	−4.142				0.723
Allele C (+)	0.233	0.015	−0.086	0.115	0.773
Allele G (+)	0.160	0.021	−0.079	0.122	0.673
Se [mg/L]	1.097	0.147	−0.174	0.468	0.368
IL-β [pg/mL]	3.490	0.791	−0.331	1.913	0.167
TNF-α [pg/mL]	1.903	0.121	−0.859	1.102	0.808
IL-6 [pg/mL]	−0.073	−0.007	−1.013	1.000	0.990
IL-β [pg/mL]*Se [mg/L]	−0.062	−0.056	−1.104	0.993	0.917
TNF-α [pg/mL]*Se [mg/L]	−0.105	−0.066	−1.074	0.942	0.898
IL-6 [pg/mL]*Se [mg/L]	−0.329	−0.746	−1.896	0.405	0.204

b—unstandardized regression coefficient; β—standardized regression coefficient; CI—confidence interval.

**Table 16:** Model II with moderation by the alleles.

	b	β	−95% CI	+95% CI	*p*-Value
Intercept	2.810				0.510
Allele C (+)	1.028	0.065	−0.281	0.412	0.712
Allele G (+)	0.710	0.095	−0.262	0.453	0.601
Se [mg/L]	0.310	0.042	−0.055	0.138	0.398
IL-β [pg/mL]	0.868	0.197	−0.052	0.446	0.121
TNF-α [pg/mL]	0.607	0.039	−0.191	0.268	0.740
IL-6 [pg/mL]	−0.803	−0.072	−0.328	0.184	0.581
Allele C*IL-6 [pg/mL]	−0.090	−0.011	−0.386	0.364	0.953
Allele C*TNF-α [pg/mL]	0.275	0.025	−0.315	0.365	0.887
Allele C*IL-β [pg/mL]	−0.517	−0.133	−0.426	0.161	0.375
Allele G*IL-6 [pg/mL]	−0.781	−0.168	−0.437	0.100	0.219
Allele G*IL-β [pg/mL]	0.364	0.128	−0.047	0.303	0.150
Allele G*TNF-α [pg/mL]	−0.134	−0.020	−0.298	0.257	0.885

b—unstandardized regression coefficient; β—standardized regression coefficient; CI—confidence interval.

## Data Availability

Not applicable.
